# Identification of contrastive and comparable school neighborhoods for childhood obesity and physical activity research

**DOI:** 10.1186/1476-072X-5-14

**Published:** 2006-03-30

**Authors:** Xingyou Zhang, Katherine Kaufer Christoffel, Maryann Mason, Lin Liu

**Affiliations:** 1 The Robert Graham Centre for Policy Studies in Family Medicine and Primary Care, American Academy of Physicians, 1350 Conneticut Avenue, NW, Suite 201, Washington, DC 20036, USA; 2 Mary Ann and J. Milburn Smith Child Health Research Program, Children's Memorial Research Center, 2300 Children's Plaza, Box157, Chicago, IL 60614, USA; 3 Department of Pediatrics and Preventive Medicine, Feinberg School of Medicine, Northwestern University, 303 East Chicago Avenue, Chicago, IL 60611-3008, USA; 4 Department of Geography, University of Cincinnati, Cincinnati, OH 45221-0131, USA

## Abstract

The neighborhood social and physical environments are considered significant factors contributing to children's inactive lifestyles, poor eating habits, and high levels of childhood obesity. Understanding of neighborhood environmental profiles is needed to facilitate community-based research and the development and implementation of community prevention and intervention programs. We sought to identify contrastive and comparable districts for childhood obesity and physical activity research studies.

We have applied GIS technology to manipulate multiple data sources to generate objective and quantitative measures of school neighborhood-level characteristics for school-based studies. GIS technology integrated data from multiple sources (land use, traffic, crime, and census tract) and available social and built environment indicators theorized to be associated with childhood obesity and physical activity. We used network analysis and geoprocessing tools within a GIS environment to integrate these data and to generate objective social and physical environment measures for school districts. We applied hierarchical cluster analysis to categorize school district groups according to their neighborhood characteristics. We tested the utility of the area characterizations by using them to select comparable and contrastive schools for two specific studies.

**Results:**

We generated school neighborhood-level social and built environment indicators for all 412 Chicago public elementary school districts. The combination of GIS and cluster analysis allowed us to identify eight school neighborhoods that were contrastive and comparable on parameters of interest (land use and safety) for a childhood obesity and physical activity study.

**Conclusion:**

The combination of GIS and cluster analysis makes it possible to objectively characterize urban neighborhoods and to select comparable and/or contrasting neighborhoods for community-based health studies.

## Background

An important decision when planning community-based health studies includes the choice of representative communities or neighborhoods. The communities selected for study will need to be few in number (for logistical and financial reasons), and must meet defined characteristics determined by the health condition under study (e.g. high or low disease rates), and the specific question being addressed (and related variables). The factors that will affect the selection of contrastive and/or comparable communities include the objectives of community program initiatives, the availability of community health indicators and health outcomes of interest, and the specific factors associated with those indicators and outcomes. Thus, research on community-based health programs requires methods for selection of representative communities or neighborhoods for interventions and investigation.

In our research, we have undertaken to detect the effects of school neighborhood social and built environments on child obesity and physical activity (and on associations between these). For this work, we seek to compare the environments of children attending defined groups of schools. The schools chosen for study are expected to be representative of groups of schools defined by environments and obesity rates, i.e.: 1) their social and built environments are contrastive between the selected school neighborhood groups and comparable within these groups and 2) their childhood obesity rate and/or physical activity levels are significantly different between neighborhood groups and are relatively similar within groups.

To achieve this, we needed to develop methods for objective characterization of neighborhood environments and to select representative neighborhoods or communities. In our large urban area, this was challenging.

In this paper, we report how we combined GIS and hierarchical cluster analysis to select contrastive and comparable school neighborhoods in Chicago for our pilot studies on child overweight and physical activity and school social and built environments.

## Results

**Figure 1:**
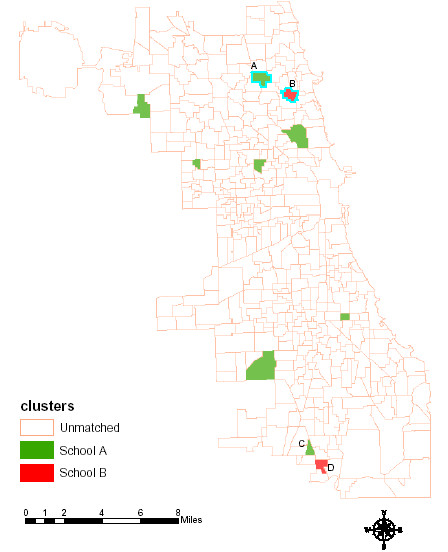
**Chicago elementary school neighborhood cluster matching with BMI data**. Showed in green are the matched schools to school A with a lower obesity rate Showed in red are the matched schools to school B with a higher obesity rate Showed in white (blank) are the unmatched schools to bother school A and B

We successfully characterized all 412 public elementary school neighborhoods. In our first application of the school neighborhood characterizations, we identified two contrastive schools on the north side Chicago in terms of overweight rate: school A and school B (Figure 1). According to Chicago Public Schools' policy, we only used the symbol names for school neighborhoods. In cluster analysis, the number of matching candidates for each school increases when the number of clusters decreases. Figure 1 shows the matching result when the cluster number was set to 150: there are 7 candidates for School A, but only one for school B: School D. School C, one of the matches for School A, is a good geographic match to School D. Although we didn't have the obesity rate for school C and D, we could expect a lower obesity rate in school C and a higher obesity rate in school D according to their school neighborhood environmental similarity to school A and B respectively.

In our second application of the utility of the school neighborhood characterizations, we undertook to define contrastive neighborhoods based only on demographic and environmental factors, e.g. using some indicators from a few variable different categories: race and ethnicity, landuse, crime and traffic. Four schools on the south side of Chicago (Table 1) were selected: E, F, G and H. Almost all students in E and G are black/African American while most students in F and H are Hispanic. (This extreme racial and ethnic composition is related to Chicago's well-documented residential segregation.)

**Table 1:** The profiles of the selected school neighborhoods.

	School	E	F	G	H
Race and Ethnicity	White (%)	0.00	0.40	0.00	3.20
	Black/African American (%)	98.70	0.30	99.90	3.40
	Hispanic (%)	1.30	99.20	0.10	92.80
Land use	population density (per acre)	6.84	16.30	24.83	39.94
	block density (per acre)	0.19	0.08	0.28	0.20
	residential (%)	18.91	18.68	75.07	82.26
	commercial (%)	8.25	1.84	3.70	13.16
	industrial (%)	1.79	53.19	0.00	0.00
	distance to park (miles)	0.40	0.36	0.13	0.21
Traffic	AADT	33100	15700	33100	25500
Crime	1997 violent crime rate (per 100000)	4482.4	651.2	2247.8	792.6

**Figure 2:**
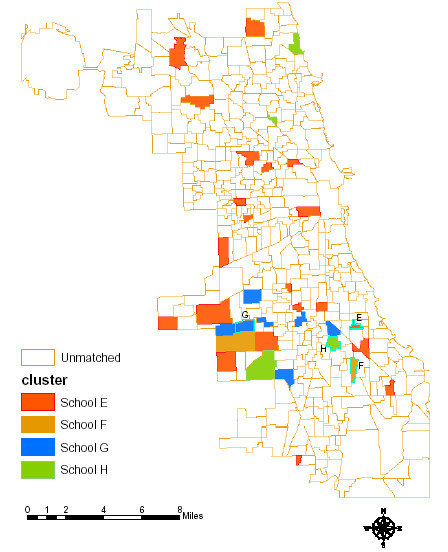
**Chicago elementary school neighborhood cluster matching without BMI data**. Showed in red are the matched schools to school E with a higher percentage of black and African American population, low population density, and low residential percentage, heavier traffic and high violent crime rate. Showed in brown are the matched schools to school F with a higher percentage of Hispanic population, low population density, and low residential percentage, lighter traffic and lower violent crime rate. Showed in blue are the matched schools to school G with a higher percentage of black and African American population, higher population density, and higher residential percentage, heavier traffic and high violent crime rate. Showed in green are the matched schools to school H with a higher percentage of Hispanic, higher population density, and higher residential percentage, lighter traffic and lower violent crime rate. Showed in white (blank) are the unmatched schools to school H, P, Z and T.

Next we did cluster analysis and identified the comparable neighborhoods corresponding to these four contrastive school neighborhoods (Figure 2). School E and F have lower population density, block density, less residential area, and longer distance to the public parks; while G and H have higher population density, block density, larger residential area, and easier access to the public parks. Both E and G have higher violent crime rate and traffic volume, while F and H have much lower crime rates and traffic volume. Thus, these four schools have distinct different races and ethnicity, landuse configuration and safety environment. This comparison maximized contrast and also included one to maximize comparability.

## Discussion

Comparison neighborhoods or communities are often used in the design of public health and epidemiology studies, in order to clarify the roles of environmental and demographic risk, and the effectiveness of community level interventions. This paper describes how we created school district characterizations and used them to identify contrastive and comparable school neighborhoods for community-based childhood obesity and physical activity studies. We used GIS to link multiple data sources to generate objective environmental measures for the school neighborhoods in Chicago, and conduct hierarchical cluster analysis to select the desirable school neighborhoods for health study. Using a combination of GIS-supported neighborhood characterization and cluster analysis, we successfully identified contrastive and comparable neighborhoods for our child physical activity and obesity research in Chicago, a large urban area.

These methods can be applied to in other urban settings to allow objective characterization of neighborhoods and the efficient and effective identification of contrastive and comparable neighborhoods for community-based health studies.

Most previous studies have been based on simple standards to select comparison neighborhoods, such as ethnic/racial mix[1], poverty level, urbanity in large-scale environmental settings (urban, suburban and rural area [2]), or the health intervention levels[3-6]. O'Camp et al. have presented the regression and principal component analysis (PCA) approaches to the identification of neighborhoods as intervention and control sites for community-based programs[7]. These methods required that health outcomes be available in all neighborhoods. For obesity and physical activity research, local community or neighborhood-level health outcomes are often not available.

We faced a large number of potential neighborhoods in Chicago, and a long list of neighborhood variables related to child physical activity and obesity. In this context, the selection of contrastive and comparison neighborhoods for the assessment of neighborhood effects on child health became quite complex. If one environmental factor has multiple indicators, factor analysis could be used to reduce data dimensions.

## Conclusion

In conclusion, this study shows a powerful methodology to select contrastive and/or comparable neighborhoods for community-based health studies and implement a logistically feasible and statistically valid sampling strategy. It appears that the combination of GIS and statistical tools provides a powerful approach to characterize neighborhood social structural context and built environment to facilitate community health programming and design.

## Data and methods

### Overview

In this report, we illustrate the methods that we have developed in applications to childhood obesity. To understand the applications, a bit of background about this condition is useful. Childhood overweight and obesity is an increasing public health problem and well known to have significant impact on both physical and psychological health. Childhood obesity has risen to unprecedented levels [8-11]. The United States 1999–2002 National Health and Nutrition Examination Survey (NHANES) indicates that, among children aged 6 through 19 years, 31.0% were at risk for overweight or overweight and 16.0% were overweight[8]. The high levels of overweight among children become a major public health concern. Overweight and obesity are assumed to be the results of a decrease in physical activity and an increase in food intake. Environmental factors may play pivotal roles in the markedly rising prevalence of obesity in the last 2 decades. Social environment, such as the poverty associated with race/ethnicity, and the built environment, including landuse patterns, transportation network and community design features, are important for obesity prevention, as they may encourage or discourage physical activity and healthy food intake [12-19]. Thus population-based obesity prevention, especially for children, may be achieved through a variety of interventions targeting built environment relevant to physical activity and diet[17].

**Figure 3:**
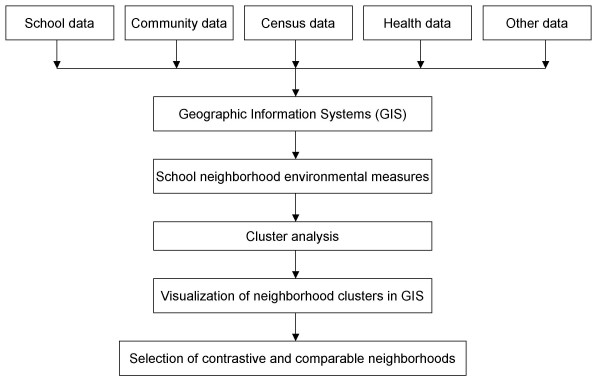
The work flow chart of selection of contrastive and comparable neighborhoods.

GIS provides a desirable digital environment to manipulate and manage a variety of data sources to characterize human subjects and neighborhood environments related to childhood obesity and physical activity. These data are represented in GIS as different layers in three formats: point (such as residence addresses, school sites, and subway stations), line (such as street networks) and polygon/area (such as neighborhood units, census geographic units such as block, census tracts). We classify the GIS data related into two categories: those related to human subjects' location as subject layers (such as home or school locations), and those related to neighborhood environment as environmental factor layers (such as land use and traffic). The neighborhood level environmental measures of interest are often not available directly and so not ready for use. In our case, the environment measures are not available at school neighborhood levels. We followed three basic steps to generate the neighborhood measures in GIS and characterize the neighborhood for our childhood obesity and physical activity studies (Figure 3).

First we used GIS to integrate publicly available social and physical environmental data from multiple sources to generate objective neighborhood environment indicators. Those indicators were selected because they are considered to be potentially associated with school children's physical activities in Chicago. This process involved spatial operations between subject layers (school neighborhoods) and environmental factor layers within GIS, using appropriate spatial imputation algorithms for different types of environmental indicators. Here spatial imputation algorithms are the procedures for the partition and/or aggregation of spatial referenced data to generate the neighborhood level measures.

Second, we combined information from a Chicago public school population survey [20], school child overweight data [21], and 2000 census data to select contrastive school neighborhoods in terms of child overweight rates, built environment and/or sociodemographics (depending on what data were available). This yielded schools that were contrastive on the variables of interest.

Third, hierarchical cluster analysis was applied to identify comparable school neighborhoods corresponding to the selected contrastive ones in terms of their social and built environments. In the following sections, we first describe the data we used, then we illustrate in detail the generation of neighborhood measures of interest and cluster analysis for identifying comparable and/or contrastive neighborhoods.

### Data Sources

#### Neighborhood unit

Like all other neighborhood- or community- based studies, we first need define the neighborhood units. In our studies, Chicago local public elementary school attendance/catchment areas are defined as the school neighborhood unit. Publicly available social and environmental data are in varied formats and from several sources (Table 2) and are not ready to use for these school neighborhood units. Therefore, social and built environment indicators have to be projected to these school neighborhood units. Table 2 lists the health outcome and neighborhood built and social environment data used for our studies.

**Table 2:** Data sources for school neighborhood characterization.

**Items**	**Factors**	**Spatial Unit**	**Data Sources**
Neighborhood Unit	School attendance boundary	School	CPS (Chicago Public School Board)
Health Outcome	BMI	School	CLOCC(Consortium to Lower Obesity in Chicago Children)
	Land use	Patch of various sizes	NIPC (Northern Illinois Planning Commission)
	AADT(Average Annual Daily Traffic)	Street segment	IDOT(Illinois Department of Transportation)
Built Environment	Public park, playground	Patch of various sizes	Chicago Department of Information and Business Service
	Bike routes	Line	
	Subway stations	Point	
	Violent crime rate	Community area	Chicago Police Department
Social Environment	Student race/ethnicity	School	CPS
	Sociodemographics	Block/tract	US Census 2000

#### Health outcome

Health outcomes for childhood obesity and physical activity data are usually not available. We have limited children's obesity data in term of Body Mass Index (BMI) data from public school children health exams [21]. BMI is calculated as weight in kilograms divided by the square of height in meters. Chicago Public School (CPS) student height and weight data on 1208 3–7 year olds were collected from 25 schools in 19 different Chicago community areas. The 2000 Centers for Disease Control and Prevention Growth Charts for the United States were used to define overweight and at risk for overweight. The Growth Charts are sex- and age-specific and are based on national data (1963–1994). At risk of overweight is defined as between the 85^th ^– 94^th ^percentiles for sex- and age-specific BMI. Overweight is defined as ≥ 95^th ^percentiles for sex- and age-specific BMI. The prevalence of overweight children was 23%, and the prevalence of children at risk of overweight 15% [21].

#### Neighborhood environment

The majority of environmental measurements in physical activity research are subjective and/or survey-based [22]. In order to characterize the neighborhood more accurately, we selected multidimensional and multilevel environmental variables relevant to child physical activity that can be measured objectively. Here we considered two general categories of environmental factors: built environment and social environment.

#### Built environment

For the built environment, we started with factors associated with adults' participation in physical activity that are already reported in the literature [23,24], including *land use, accessibility*, and *neighborhood safety*.

##### Land use

Land use refers to the spatial distribution of human activities in a defined space. The major land use types in an urban neighborhood include residential, commercial, industrial, institutional and others. *Land use composition, diversity, and fragmentation *are among its basic dimensions. Land use composition is described by the percentages of each land use types in a neighborhood. For example, a residential neighborhood could have more than 50% area classified as residential while in Chicago downtown will have more than 50% area as commercial. We used the number of land use types in a neighborhood to reflect the land use mix, and the number of land use patches to indicate land use fragmentation. Urban neighborhood land use configuration is associated with physical activity or travel behavior and a mixed land use in a neighborhood (locating different types of activities close together, such as shopping stores and schools within or adjacent to residential neighborhoods) may promote residents' physical activity[19,25]. We generated 12 land use types for all areas within the school neighborhoods, including residential, commercial, industrial and urban open space.

##### Accessibility

Accessibility often refers to the spatial access to or from destinations or facilities in a neighborhood. Street density is a common measure for accessibility. Since Chicago is located on a flood plain, with a well developed grid street system, block density is closely related to street density and is an appropriate indicator of accessibility associated with pedestrian travel behaviors[26]. We extend Eash's use of census blocks to use the block density in each school neighborhood to measure neighborhood accessibility as well as the pedestrian environmental suitability[26]. For children, the proximity to public parks and playgrounds was used to assess accessibility of public areas for play or exercise. We derived the accessibility of schools to public parks and school playgrounds in term of distance for each school neighborhood.

##### Safety

Neighborhood safety in Chicago is significantly associated with the reduced children's physical activity level[27]. According to our pilot focus group data (from a project called Transportation is Active and Safe for Kids (TASK)[28]), safety related to both crime and traffic is the major concern of Chicago parents in allowing children to walk to school. Although schools are not far from homes and most elementary schools have an attendance boundary with a radius of less than half mile, many parents hesitate to let children walk to school alone due to fear of crime and/or wide and busy streets. Other studies also show that neighborhood safety is important for children's physical activity [29-31], more so than for adults' physical activity [23]. We assessed two major aspects of urban neighborhood safety: *traffic *and *violent crime*.

##### Traffic

Heavy urban neighborhood traffic in Chicago is a serious threat to school children and a major cause of injury to them [29]. Most methods for acquiring neighborhood traffic information in larger urban areas were infeasible for our needs. For example, Chicago has 24,749 census blocks, 876 census tracts (within 77 Chicago community areas); as a result, direct field traffic survey is very difficult, both physically and economically. We adopted two indicators to measure the school neighborhood traffic status: 1) the number of arterial streets within local school areas and 2) the maximum average annual daily traffic (AADT), a number of vehicles on the arterial streets. We did not include the interstate highway passing by school neighborhood for traffic assessment, as the traffic on interstate highway are quite isolated from the local street systems.

##### Crime

Neighborhood crime events, especially violent crime events (homicide, aggravated assault, robbery and criminal sexual assault) may be a significant environmental barrier to outdoor physical activity and affect neighborhood safety perceptions [22,32,33]. A twenty-year Chicago violent crime study shows that neighborhood violent crime rates are quite stable over years[34]. We used Chicago community area violent crime rates for incidents involving youth victims as our measure of neighborhood crime vulnerability [35].

##### Social environment factors

Neighborhood socioeconomic status (SES), such as poverty [36], education and employment are fundamental factors that influence health and well-being [37], including child overweight and physical activity [32,38]. In Chicago, one of the most residentially segregated cities in the US, neighborhoods' physical layouts (street design) are closely related to their socioeconomical status. We selected census tract level data on racial and ethnic composition, educational attainment, unemployment and poverty rates to measure school neighborhood social and economic status.

#### Spatial analysis in GIS

We used GIS to generate school-level neighborhood environmental indicators from those originally created for other spatial units (e.g. census tract, street segment, and Chicago community area). Two spatial operations in GIS were used: *spatial partition *and *spatial aggregation*. *Spatial partition *here is defined as the process of linking environmental GIS layers with school boundary layer according to their spatial relationships. *Spatial aggregation *is the process of summarizing environmental measurements at the school level. Since the school neighborhood is a GIS layer in format of polygon or area, there are three types of spatial partition and aggregation between school GIS layer and environmental GIS data layers: polygon-point, polygon-line, and polygon-polygon.

If the original environment data are represented in point format in GIS, the spatial partition and aggregation are quite intuitive. For example, the block density is defined as the number of how many blocks per school neighborhood. Since a block centroid is only within only one school neighborhood, we assigned it to a school neighborhood, and then summarized the number of block centroid points within a school neighborhood, we divided the number of block centroid points by the school neighborhood area to generate the block density for each school neighborhood.

If the original environment data are represented in line format in GIS, spatial partition and aggregation are similar to those applied to point data. For example, neighborhood traffic was based on AADT on arterial street segments. We could assign the whole street segment or part street segment to a school neighborhood and then we perform the necessary spatial aggregation to generate the indicators of interest for each school. We obtained the number of arterials and maximum AADT for each school neighborhood.

If the original environment data are represented in polygon (or area) in GIS, the spatial partition and aggregation are a little more complex. We illustrate this in detail using the case of census tract to generate school neighborhood socioeconomic indicators.

**Figure 4:**
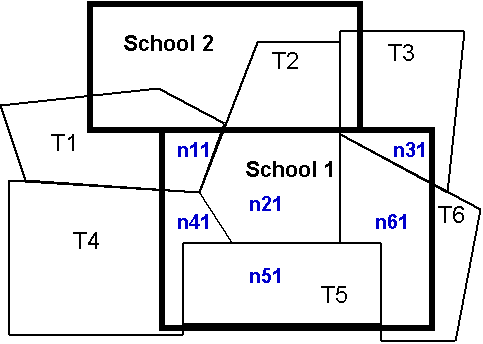
**The spatial relationship between school neighborhoods and census tracts**. Public elementary school boundaries are shown in wide and thick bold line (school 1 and 2). Census tract boundaries are shown in single and thin line (census tract 1 to 6) *n*_*ij *_are newly created polygon within school j from census tract i

• *Spatial Partition*: 412 public elementary schools with attendance boundaries were overlapped with census tract boundaries to create a new polygon layer in GIS. In this newly created GIS layer, a school neighborhood was divided into multiple polygons; and a census tract was often split into multiple polygons if the tract is across a school neighborhood boundary and belongs to more than one school. Figure 4 shows a school neighborhood (school 2) that included multiple polygons (n1–n6) from different census tracts (T1–T6); on the other hand, a census tract (T2) may belong to multiple school neighborhoods (school 1 and school 2). Within GIS, both school and census tract identifiers are assigned to all new formed polygons (e.g. n11–n61) and their areas are recalculated. Each polygon remains then linked with its census tract sociodemograhic data by its unique census tract unit identifier.

• *Spatial Aggregation*: SES data were aggregated for school neighborhoods based on the census tract segments generated above. Two different algorithms for spatial imputation were used to calculate the school-level indicators from census tract-level ones. Sociodemographic measures were assumed to be evenly distributed within a census tract. Specifically, suppose that *a*_*ij *_is the area of a newly created polygon *n*_*ij *_within school j from census tract i, the area for school j, , and the area for census tract i, . Suppose *r*_*i *_is one SES indicator for tract i, then the aggregated SES indicator for school j, *p*_*j*_, is 1) the summary of *r*_*i *_, weighted by the proportion of the area of a newly created polygon within the school,  if *r*_*i *_is non-summable indicator (e.g. family median income), or 2) the summary of *r*_*i*_, weighted by the proportion of the area of a newly created polygon to its census tract,  if *r*_*i *_is summable indicator (e.g. population). For example, consider a school neighborhood with an area of 100 acres composed of only three census tracts: one tract with a population 2000 and family median income $35,000 has an area 20 of 40 acres within the school neighborhood, the other tract with a population 3,000 and family median income $45,000 has an area 30 of 50 acres within the school neighborhood, and the last tract of 50 acres with a population 1,500 and family median income $40,000 is nested in the school neighborhood. Now we need generate the school neighborhood family median income and population. Family median income is a non-summable indicator, and according to the first summary formula above, the neighborhood family median income equals to 40500(35000*20/100+45000*30/100+40000*50/100). Since Population is summable indicator, and according to the second summary formula, the neighborhood population equals to 43000(2000*20/40+3000*30/50+1500*50/50).

#### Cluster analysis

Cluster analysis was used to group school neighborhoods by characteristics of social and built environments. There are 412 public elementary school neighborhoods in Chicago. In order to select representative neighborhoods for our pilot studies, it is necessary to characterize these school neighborhoods into a small number of categories (clusters) according to the similarity of their neighborhood environments:

• First, we used public elementary school health exam data on 19 schools to identify 2 contrastive schools in terms of school student overweight rate. These schools had relatively larger sample sizes (N = 180, 113); one with a lower obese rate (20.6%) and the other with a higher one (33.3%). The schools are quite close to each geographically; one has > 90% Hispanic student, and the other has 50% black and 50% Hispanic students.

• Second, we used school demographic survey data (Chicago Public School 2001) and school neighborhood SES indicators from US census 2000 to identify schools similar to these two, based on the percentages of white, black, and Hispanic student from the 2001 Chicago public school survey to represent student population demographic profiles. We also included the environmental indicators listed in Table 1 and 3 in the cluster analysis. Since indicators with large variances tend to have a larger effect on the resulting clusters than those with small variances, we first standardized all variables prior to cluster analysis [39] to generate the neighborhood cluster hierarchical cluster trees. Cluster analysis was implemented in SAS.

**Table 3:** The selected socioeconomic indicators for school neighborhoods.

Factor	Indicators	Unit
Educational attainment	Population 25 years and over with high school graduate or over	percent
wealth status	average family median income	number
social stability	unemployment rate for population 16 years and over	percent
cultural context	region of birth of foreign born	percent
poverty level	the poverty rate of family with children under 5 years	percent

• Third, we visualized the cluster analysis results in a map within GIS, which facilitated the selection of school neighborhoods that were not only environmentally contrastive and comparable, but also geographically close.

## Competing interests

The author(s) declare that they have no competing interests.

## Authors' contributions

XZ, KKC conceived the study and wrote the final version of the paper

MM prepared the school obesity data and participated in the draft revision.

LL provided GIS technical support and participated in the draft revision.
